# The Presence of a Parasite in the Head Tissues of a Threatened Fish (*Bidyanus bidyanus*, Terapontidae) from South-Eastern Australia

**DOI:** 10.3390/pathogens12111296

**Published:** 2023-10-30

**Authors:** Diane P. Barton, R. Keller Kopf, Xiaocheng Zhu, Shokoofeh Shamsi

**Affiliations:** 1 School of Agricultural, Environmental and Veterinary Sciences, Charles Sturt University, Wagga Wagga, NSW 2650, Australia; sshamsi@csu.edu.au; 2 Gulbali Institute, Charles Sturt University, Wagga Wagga, NSW 2650, Australia; 3 Research Institute for the Environment and Livelihoods, Charles Darwin University, Darwin, NT 0801, Australia; keller.kopf@cdu.edu.au; 4 Wagga Wagga Agricultural Institute, NSW Department of Primary Industries, Wagga Wagga, NSW 2650, Australia; xiaocheng.zhu@dpi.nsw.gov.au

**Keywords:** behaviour, conservation management, digenetic trematode, Murray-Darling Basin, parasite-host interaction

## Abstract

The silver perch, *Bidyanus bidyanus* (Mitchell) (Terapontidae) is a freshwater fish, endemic to the Murray-Darling river system in south-eastern Australia. Population declines have led to the fish being listed as critically endangered by the Australian Government. Knowledge about parasites and diseases of wild populations of freshwater fish are limited in Australia. During an examination of wild-caught silver perch, digenean mesocercaria were observed in the head tissues. A total of five of the 11 silver perch collected from the Wakool River, New South Wales, were infected with mesocercaria. All mesocercaria were found in the head tissues; no mesocercaria were found encysted in the eye lens. The mesocercaria were found to belong to the family Strigeidae based on the sequences of their internal transcribed spacer (ITS) region. The lack of comparable sequences of strigeid digeneans from Australian hosts precludes being able to determine if the mesocercaria found in this study are a new species or representatives of an already described species. However, genetic results confirm that this is a different species to other digeneans previously described from silver perch, thus increasing the number of digeneans reported from *B. bidyanus* to three species. The presence of digenean mesocercaria in the head tissues of a wild population of silver perch, as found in the present study, is of potential conservation significance. Given the critically endangered conservation status of *B. bidyanus*, and previous evidence of strigeid infection altering fish behaviour, ecology, and predation mortality, further research on the potential impacts of infection on wild populations is warranted.

## 1. Introduction

The silver perch, *Bidyanus bidyanus* (Mitchell) (Terapontidae) is a freshwater fish, endemic to the Murray-Darling River system in south-eastern Australia [1]. Due to severe declines in abundance and distribution, silver perch are listed as critically endangered by the Australian Government [2]. Silver perch are caught in recreational fisheries, are food fish produced in aquaculture, and are produced in hatcheries for fisheries stocking and conservation purposes [3]. The causes of population declines have largely been attributed to river regulation [4] and competition with invasive carp (*Cyprinus carpio*) [5], but the potential impacts of parasitic diseases on species conservation status remain unexplored.

Parasites can alter host behavior and ecology but their role in regulating individual, population, and community dynamics is often ignored in fish biology and conservation research [6,7,8]. Most of our knowledge about parasites of silver perch is from aquaculture systems, where protozoans (*Chilodonella* spp., *Ichthyobodo necator*, *Ichthyophthirius multifilis*, *Tetrahymena* sp. and *Trichodina* sp.) and ectoparasitic monogeneans (*Lepidotrema bidyani* Murray) have accounted for approximately 80% of the reported diseases of silver perch; no digeneans have been reported yet [9]. Knowledge about parasites of wild populations of silver perch is extremely poor, with no reports for over 40 years [10]. Wild-caught fish have been reported with a small range of parasites, including *L. bidyani*, four species of intestinal nematodes, and two species of digeneans [11]: an adult *Pretestis australianus* Angel & Manter from the intestinal system [12] and a metacercarial (larval) stage of *Diplostomum spathaceum* (Rudolphi) from the eye [13]. The latter record is interesting because digeneans belonging to the Diplostomatoidae, including *D. spathaceum*, are well-known as parasites in fish elsewhere in the world, but rarely reported in Australia. It is known that this infection leads to blindness in the fish which may impact the ecology of the host through differential predation by birds, the presumed definitive hosts [14]. Infections of metacercaria in the brain of various species of fish have been reported elsewhere, leading to physical abnormalities [15] and/or alterations in behavior that can lead to ecological impacts at host individual, population or community levels [6].

There are a few reports of digenean metacercaria in the heads of fish in Australia [16,17] and the ecological and conservation impacts of infection remain unknown. As with many host groups in Australia, the study of parasites in wild freshwater fish is limited [10,18]. The aim of the present study is to provide information on the occurrence and identification of a previously unknown digenean larval stage observed in the head tissues of wild-caught silver perch.

## 2. Materials and Methods

As part of a project on fish behavior, a total of 11 silver perch (mean total length 151.5 mm (113–212 mm)) were collected from the Wakool River, New South Wales, in the Murray-Darling basin between 4 and 27 February 2019. At the completion of the experiments, the fish were immediately euthanized with a benzocaine solution and frozen in individual plastic bags. The fish were then partially defrosted and a dorsal incision was made at the top of the head for removal of the otoliths for ageing (10 fish were determined to be within the first year; one fish was determined to be in the second year). The fish carcasses were then refrozen and subsequently donated to the Parasitology Laboratory, Charles Sturt University, Wagga Wagga, for parasitological examination. During the dissection process, and the removal of the gills, digenean larval stages were found in the petri dish. Subsequent exploration found that the larval stages were coming from within the head of the fish; due to the otolith incision, the exact location of origin could not be determined, but most larval stages were found in the cranial cavity.

Larval stages were collected and preserved in 70% ethanol. A number of specimens had a small section excised for molecular analysis prior to processing for morphological analysis. Specimens were rehydrated, stained with aceto-orcein, dehydrated through a series of ethanol concentrations, cleared in xylene, and mounted in Canada balsam. Specimens were studied morphologically by light microscopy (Nikon, Sydney, Australia) and the few characters of systematic importance present measured. Specimens were identified using the keys of Niewiadomska [19,20]. Specimens have been deposited in the Australian Museum. All measurements are given in micrometers, unless stated otherwise. Mean measurements are given, followed by the range in parentheses. Photographs were taken using a 9-MP microscope digital camera (AmScope Model MU900, Irvine, CA, USA).

Genomic DNA was isolated from selected larval samples (n = 5) using DNeasy Blood & Tissue Kits (Qiagen, Melbourne, Australia) with some modifications in the manufacturer’s protocol [21] and eluted into 45 μL of water. The nuclear ITS1 region was amplified using the primer pair D1F (5′-AGGAATTCCTGGTAAGTGCAAG-3′) and 5.8S Diplostomum_R990 (5′-ATCCCGCGGCCGCAATAT-3′), while the ITS2 region was amplified using primer pair 5.8S_Diplostomum_F838 (5′-TCTGAGCGGTGGATCACTC-3′) and D2R (5′-CGTTACTGAGGGAATCCTGG-3′). Primers D1F and D2R were designed by Hillis & Dixon [22] while primers 5.8S_Diplostomum_F838 and 5.8S Diplostomum_R990 were designed in this study. PCR cycling condition was 2 min of initial denaturing at 95 °C, followed by 40 cycles of 30 s of denaturing at 95 °C, 30 s of annealing at 50 °C, and 1 min of extension at 72 °C. The PCR reaction was completed by a final extension for 10 min at 72 °C. PCR amplicons were sent to the Australian Genome Research Facility (AGRF Ltd., Brisbane, Australia) in Queensland for bidirectional sequencing using the same primers. Forward and reverse AB1 trace files were quality checked using SeqMan v.8.1.0. The ITS1 and ITS2 sequences were aligned and the contig was used for the downstream analysis.

ITS sequences of members of the Strigeinae subfamily were obtained from GenBank and a *Uvulifer* Yamaguti (Diplostomidae) species was used as an outgroup (Supplementary Table S1). Sequences were aligned with our sample using BioEdit v 7.0.9.0 [23]. Pairwise genetic distances were calculated using MEGA X [24]. The phylogenetic analysis was performed using MrBayes 3.2 [25] for 1,500,000 generations until the average standard deviation was lower than 0.005, using the GTR + G model as inferred by jModelTest 2 [26]. Alignment gaps were excluded from analyses.

## 3. Results

A total of five of the 11 (45.5%) silver perch were found to be infected with digenean larval stages, with a mean intensity of infection of 3.8 (range 1–7). All larval stages were found in the head tissues or the petri dish; no larval stages were found encysted in the eye lens.

### 3.1. Morphology of Larval Stage

**Figure 1:**
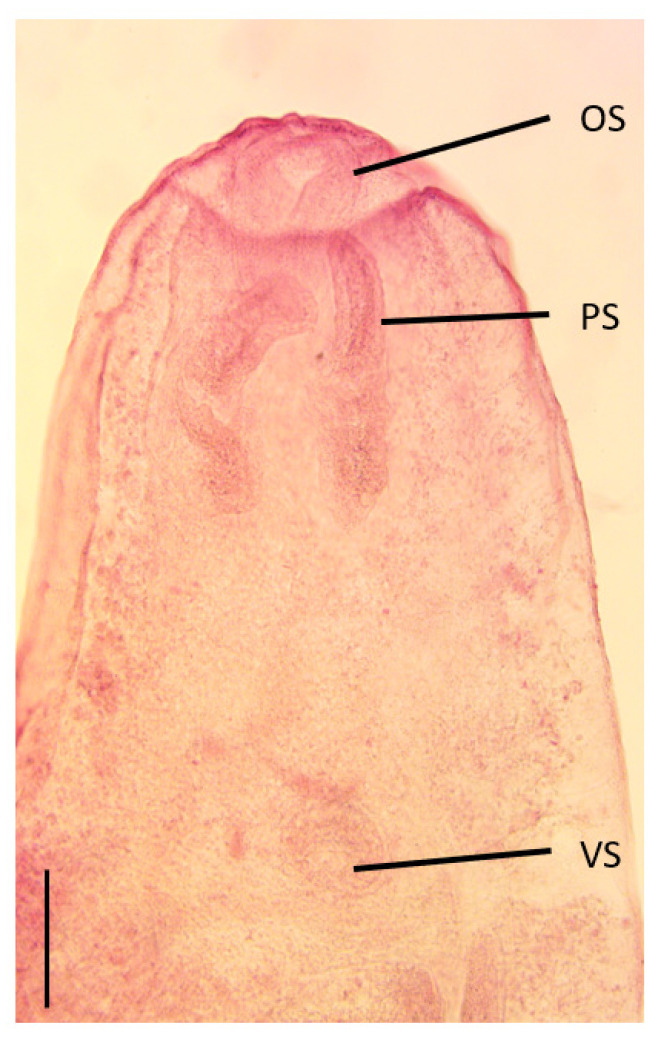
Photograph of the anterior end of the specimen of digenean mesocercaria collected from the head tissues of silver perch, *Bidyanus bidyanus*, in this study. OS, oral sucker; PS, pseudosucker; VS, ventral sucker. Scale bar 200 µm.

Description of larval stage (n = 7) (Figure 1): Body ovoid, 1650 (1475–1825) (number measured 7) total length, 771.3 (687–875) (7) maximum width. Tegument smooth. Oral sucker round, slightly longer than wide; 175.7 (150–200) (7) long by 158.6 (130–210) (7) wide. The oral sucker appeared “sunken” into the oral collar in some specimens. Paired pseudosuckers are present in some specimens near the edge of the oral collar; 310 (270–350) (2) long, 110 (1) wide. The pharynx was not visible. The thin oesophagous splits into two simple intestinal caeca; the caeca run along the lateral edge of the body; the caeca appear to bend toward the midline posterior to reproductive organs; possibly with a transverse connection to make an H shape; caecae ending blind 58.3 (55–60) (3) from the posterior end of the body. Ventral sucker in the midline, approximately mid-way down the body; round 131.7 (100–160) (6) long by 140 (130–170) (7) wide. Developing reproductive organs posterior to ventral sucker in the posterior third of the body, 430 (350–550) long by 303.3 (240–420) wide (6). The excretory bladder was elongated and tubular.

#### Morphological Remarks

Due to a combination of the location within the host (in the head) and the presence of pseudosuckers, the specimens were identified as a member of the superfamily Diplostomoidea [19]. The specimens were not examined alive which prevented a description of the excretory system which is used to differentiate types of metacercaria [19]. The absence of a cyst surrounding the larval stage suggests a ‘Diplostomulum’ type of metacercaria with pseudosuckers present [19]. However, due to our method of collection, the confirmed presence or absence of a cyst is unknown. Thus, it is possible that the larval stage is either a ‘Neascus’ (usually encapsulated with pseudosuckers present or absent) or a ‘Tetracotyle’ (always encapsulated and pseudosuckers present) type [19]. It is noted, however, that mesocercarial stages, may occur in the life cycles of some species who normally have a ‘Tetracotyle’ type metacercaria [20,27]. Mesocercaria are an unencysted developmental stage, found between the cercaria and metacercaria [27]. Therefore, the larval stage collected in this study appears to be a mesocercaria. Thus, identification of the mesocercaria found in this study beyond superfamily Diplostomoidea is problematic without examination of more specimens and/or a range of genetic sequences from identified adult specimens to match against. Superficially, however, we suggest the mesocercaria belongs to the Family Strigeidae based on the presence of pseudosuckers. Adults of this family are generally found in birds, with one genus found in mammals [20].

### 3.2. Molecular Sequencing

**Figure 2:**
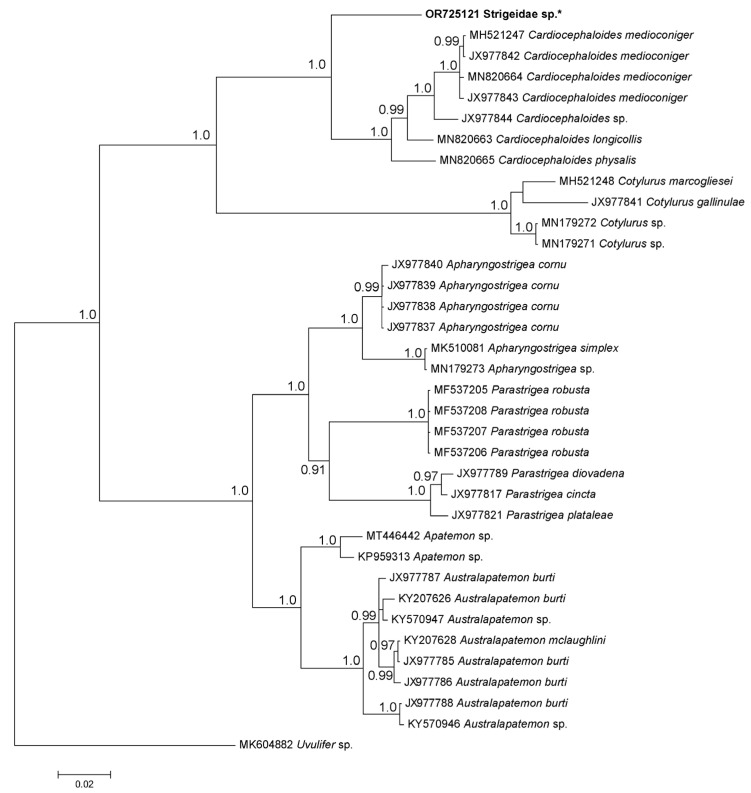
Phylogenetic tree of ITS region for mesocercaria collected from silver perch, *Bidyanus bidyanus*, in the present study (indicated in bold and with an asterisk). Posterior probabilities that are over 90% were indicated near the nodes.

PCR and sequencing were only successful in one sample. The alignment of sequences used for phylogenetic analysis was truncated to 1224 bp in length. Phylogenetic analysis (Figure 2) placed the mesocercaria from silver perch within a clade of the family Strigeidae that included the genera *Cotylurus* Szidat and *Cardiocephaloides* Sudarikov. The average genetic distance between our sample and *Cotylurus* and *Cardiocephaloides* species were 5% and 15% (Supplementary Table S2), respectively. This clade was separated into a clade that included specimens of the strigeid *Apatemon* Szidat that were collected from the body tissues of gudgeons (*Hypseleotris* sp.) in the same region [28].

#### Genetic Remarks

Metacercaria of members of the genus *Cardiocephaloides* are found in marine fish, with adults in gulls, terns, and penguins around the world [20]. All of the sequences available were from adult flukes, predominately from North and South America. Although the Wakool River is approximately 800 km inland from Sydney (on the eastern coast of Australia), there have been reported sightings of seagulls in the region [29], so it is possible that this parasite may have a marine definitive host. The other genus in the clade, *Cotylurus*, however, is associated with freshwater hosts, principally ducks [20]. Again, the sequences available on GenBank were primarily from North and South America; however, there were some sequences that had been obtained from cercaria from snails (MN179271-MN179272). Based on the lack of similarity with the available sequences, we suggest that the mesocercaria collected from silver perch in this study belongs to a different genus.

## 4. Discussion

We propose that the specimens found in the head tissues of the silver perch, *B. bidyanus*, are members of the family Strigeidae based on several factors, especially the presence of pseudosuckers and the molecular results. Larval strigeids are often difficult to identify and, in some instances, the species cannot be identified with certainty without undertaking experimental infections to retrieve the adult stage [30]. As the taxonomy of digeneans is based on the morphology of the adult, and many of the larval stages bear no resemblance to the adult, it is often difficult to link larval and adult stages [31,32]. Genetic sequencing provides confirmation of species identity of larval stages [31], but only when the corresponding adult stage has been identified and sequenced. The lack of sequences of strigeid digeneans from Australian hosts for comparison precludes us from being able to determine if the mesocercaria are a new species or a representative of an already described species.

Metacercaria of the family Strigeidae are commonly reported within the tissues of their second intermediate hosts, including fish [14,20,33]. Some strigeid digeneans have a mesocercarial stage in their life cycle in the intermediate and/or paratenic host that may not encyst [20,34]. Mesocercaria have so few characteristics, especially in fixed specimens that it is difficult to determine to which species they belong without experimental infections [35]. It is most likely that the stages collected from the silver perch in this study are mesocercaria, not metacercaria. Further study is required on fresh host specimens to determine the true location of the parasite within the host, the presence or absence of a cyst, as well as to examine specimens alive to determine the excretory system for full identification.

The life cycle of the majority of Strigeids with larval stages in fish have fish-eating birds as definitive hosts and enter fish via molluscan first intermediate hosts [20,30]. Mawson et al. [36] provided a checklist of parasites of birds in Australia, with approximately 21 species across 7 genera within the Family Strigeidae reported. Many of these waterbird hosts have wide geographical distributions across Australia, which includes the Murray-Darling river system [37]. There has been little work on the parasites of both waterbirds and freshwater fish in Australia [10,18,38] and most of their parasite faunas remain undocumented. Our sequencing results did not provide a specific identification, which is not surprising given the paucity of sequences for strigeids from Australian hosts. There are many species of strigeids reported by Mawson et al. [36] that have not had genetic sequencing completed. The mesocercaria in this study may well belong to one of those, or could, potentially, be a new species. More work on all parasites of Australian freshwater-associated fish and waterbirds is required to determine the true extent of their parasite fauna as well as potential life cycles.

The results of this study increase the number of digeneans reported from *B. bidyanus* to three species. Two digeneans have been reported from silver perch: the diplostomulum of *Diplostomum spathaceum* from the lens of the eye [11,13,39] and the adult *Pretestis australianus* (Paramphistomidae) [11,12,39] from the intestine. This is the first report of digenean mesocercaria from the cranial cavity/brain of the silver perch.

*Pretestis australianus* has been found in a range of freshwater fish species [12] across a wide geographic range in Australia [40]. However, the digenean found in the silver perch in this study were a mesocercarial stage and not a paramphistomid, so were not the same species.

The Diplostomulum of *D. spathaceum* has also been reported from a wide variety of native and introduced fish [11]; all from the eye lens. Other species of Diplostomulum have been reported from other parts of the fish body [11], although none were reported from the brain. Chapman et al. [18] reported *Diplostomum* sp. metacercaria in *Galaxias maculatus* (Jenyns) in southwestern Western Australia. These parasites caused “black spot disease” where the fish presented with small, dark, raised lesions on the skin which are the metacercaria encysted within the underlying dermis and muscle. Negm-Eldin and Davies [17] reported encysted metacercaria of the strigeid digenean *Apatemon hypseleotris* from the gudgeon, *Hypseleotris klunzingeri* (Ogilby), from the Murray River. In experimental infections of *Gambusia holbrooki* Girard, metacercaria were found encysted in the cranium, the body cavity and eye. Similarly, metacercaria of *A. gracilis*, a widespread strigeid reported across the world, were found encysted in the cranial and coelomic cavities of *Galaxiella* sp. from south-eastern Australia [16]. Metacercaria of *Apatemon* sp. were recently identified from gudgeons, *Hypseleotris* spp., close to the area where the silver perch in this study were collected [28]. These metacercaria were encysted throughout the musculature and body cavity, with none collected from the cranial cavity. Genetic sequencing showed that the metacercaria collected by Shamsi et al. [28] were not the same species as those collected from silver perch in this study.

The detection of digenean mesocercaria in the head tissues of a wild population of the critically endangered silver perch, *B. bidyanus*, is of potential conservation significance. Given the critically endangered conservation status of *B. bidyanus*, the potential impacts of strigeid infection on fish behaviour and ecology warrants further attention. Population declines of *B. bidyanus* have largely been attributed to river regulation [4] and competition with invasive carp [5]. However, these parasites may represent, depending on the pathology and prevalence of strigeid infections, an important factor limiting recovery efforts for this species. *Bidyanus bidyanus* in this study were juveniles and therefore potential recruitment to adult populations, from wild sources and hatchery releases, may be affected. As this fish species is also often a target for aquaculture, it is important to recognize that this parasite may be present. The prevalence of strigeid infection in *B. bidyanus* across different age-classes requires more comprehensive sampling.

Members of the strigeid digenean genera *Diplostomum* von Nordmann and *Tylodelphys* Diesing are important parasitic pathogens of both farmed and wild stocks of fish around the world [14,33,41]. Infections with metacercaria of *Diplostomum* and *Tylodelphys* are known to induce blindness, emaciation, cranial deformation, poor growth, and, in some species, host mortality [14,33,41,42]. Also, it is suggested that infection with metacercaria within specific lobes of the brain may affect vision and motor control [15,41], with potential consequent effects on feeding behaviour and predator escape responses [6,14,33,41]. For example, fish with infected eye lenses and brains have been known to spend more time in the open and use refuges less [42], swimming nearer the surface [43] and not shoaling [44]. These disease expressions are hypothesized to increase the chances of predation by the definitive hosts (e.g., waterbirds) and thereby complete the life cycle of the parasite. Generally, parasites are not expected to be ultimate drivers of species extinctions owing to host-parasite co-evolution. However, parasite infection can be a major source of mortality and an ultimate cause of extinction in endangered species with low population sizes [45]. It remains unknown whether this strigeid infection of *B. bidyanus* has pathological or behavioural impacts that may exacerbate population declines of this critically endangered species, but we suggest further research is needed.

## Data Availability

Whole mounted and wet specimens of the mesocercaria have been deposited in the Australian Museum. Molecular sequences have been deposited in GenBank.

## Supplementary Materials

The following supporting information can be downloaded at: https://www.mdpi.com/article/10.3390/pathogens12111296/s1, Table S1: Sequences used in sequence analysis of the mesocercaria collected from silver perch in this study [28,46,47,48,49,50,51,52]; Table S2: Genetic distance of the ITS sequences used in this study.
