# Supplementary material for: The Presence of a Parasite in the Head Tissues of a Threatened Fish (Bidyanus bidyanus, Terapontidae) from South-Eastern Australia
Source: Pathogens. 2023 Oct 30;12(11):1296. doi: 10.3390/pathogens12111296 (PMC10674335; doi:10.3390/pathogens12111296)
Supplement: Supplementary file 1 [file pathogens-12-01296-s001.zip › pathogens-2680109-supplementary.pdf]

Supplementary Table S1. Sequences used in sequence analysis of the mesocercaria collected from silver perch in this study. *Uvulifer* sp. was used as the outgroup. DS = Developmental stage: A, Adult; MC, Metacercaria. NA = Not available.

ID	Species Name	Accession Number	DS	Host species	Locality	Reference
1	Strigeidae sp.	TBC	MC	*Bidyanus bidyanus*	Wakool River, NSW, Australia	This study
2	*Cardiocephaloides medioconiger*	MH521247	A	*Thalasseus maximus*	Florida, USA	Locke *et al.* (2018)
3	*Cardiocephaloides medioconiger*	JX977842	A	*Larus* sp.	Campeche, Mexico	Hernández-Mena *et al.* (2014)
4	*Cardiocephaloides medioconiger*	MN820664	A	*Thalasseus maximus*	Mississippi, USA	Achatz *et al.* (2020)
5	*Cardiocephaloides medioconiger*	JX977843	A	*Larus* sp.	Campeche, Mexico	Hernández-Mena *et al.* (2014)
6	*Cardiocephaloides* sp.	JX977844	A	*Larus occidentalis*	Baja California Sur, Mexico	Hernández-Mena *et al.* (2014)
7	*Cardiocephaloides longicollis*	MN820663	A	*Larus argentatus*	Kyiv Oblast, Ukraine	Achatz *et al.* (2020)
8	*Cardiocephaloides physalis*	MN820665	A	*Spheniscus magellanicus*	Magallanes Region, Chile	Achatz *et al.* (2020)
9	*Cotylurus marcogliesei*	MH521248	A	*Lophodytes cucllatus*	Quebec, Canada	Locke *et al.* (2018)
10	*Cotylurus* sp.	MN179272	C	*Biomphalaria straminea*	Minas Gerais, Brazil	López-Hernández *et al.* (2019)
11	*Cotylurus* sp.	MN179271	C	*Biomphalaria straminea*	Minas Gerais, Brazil	López-Hernández *et al.* (2019)
12	*Cotylurus gallinulae*	JX977841	A	*Aythya affinis*	Sonora, Mexico	Hernández-Mena *et al.* (2014)
13	*Apharyngostrigea cornu*	JX977840	A	*Nyctanassa violacea*	Veracruz, Mexico	Hernández-Mena *et al.* (2014)
14	*Apharyngostrigea cornu*	JX977839	A	*Nyctycorax nyctycorax*	Sinaloa, Mexico	Hernández-Mena *et al.* (2014)
15	*Apharyngostrigea cornu*	JX977838	A	*Butoroides virescens*	Veracruz, Mexico	Hernández-Mena *et al.* (2014)
16	*Apharyngostrigea cornu*	JX977837	A	*Ardea alba*	Veracruz, Mexico	Hernández-Mena *et al.* (2014)
17	*Apharyngostrigea simplex*	MK510081	A	*Egretta thula*	Dero, Argentina	Unpublished
18	*Apharyngostrigea* sp.	MN179273	C	*Biomphalaria straminea*	Minas Gerais, Brazil	López-Hernández *et al.* (2019)
19	*Parastrigea robusta*	MF537205	MC	*Lissotriton vulgaris*	Koblenz, Germany	Sinsch *et al.* (2019)
20	*Parastrigea robusta*	MF537208	MC	*Lissotriton vulgaris*	Koblenz, Germany	Sinsch *et al.* (2019)
21	*Parastrigea robusta*	MF537207	MC	*Lissotriton vulgaris*	Koblenz, Germany	Sinsch *et al.* (2019)
22	*Parastrigea robusta*	MF537206	MC	*Lissotriton vulgaris*	Koblenz, Germany	Sinsch *et al.* (2019)
23	*Parastrigea diovadena*	JX977789	A	*Eudocimus albus*	Sinaloa, Mexico	Hernández-Mena *et al.* (2014)
24	*Parastrigea cincta*	JX977817	A	*Eudocimus albus*	Sinaloa, Mexico	Hernández-Mena *et al.* (2014)
25	*Parastrigea plataleae*	JX977821	A	*Platalea ajaja*	Sinaloa, Mexico	Hernández-Mena *et al.* (2014)
26	*Apatemon* sp.	MT446442	MC	*Hypseleotris klunzingeri*	Narrandera, NSW, Australia	Shamsi *et al.* (2021)
27	*Apatemon* sp.	KP959313	MC	*Percottus glenii*	Amur River, Russia	Unpublished
28	*Australapatemon burti*	JX977788	A	*Oxyura jamaicensis*	Durango, Mexico	Hernández-Mena *et al.* (2014)
29	*Australapatemon burti*	JX977785	A	*Anas americana*	Baja California Sur, Mexico	Hernández-Mena *et al.* (2014)
30	*Australapatemon burti*	JX977786	A	*Anas cyanoptera*	Estado de México, Mexico	Hernández-Mena *et al.* (2014)
31	*Australapatemon burti*	KY207626	C	*Helisoma trivolvis*	Alberta, Canada	Gordy *et al.* (2017)
32	*Australapatemon burti*	KY570947	C	Planorbidae sp.	California, USA	Gordy *et al.* (2017)
33	*Australapatemon burti*	JX977787	A	*Anas diazi*	Estado de México, Mexico	Hernández-Mena *et al.* (2014)
34	*Australapatemon* sp.	KY570946	A	*Oxyuris jamaicensis*	Lake Manitoba, Canada	Gordy *et al.* (2017)
35	*Australapatemon mclaughlini*	KY207628	C	*Physella gyrina*	Alberta, Canada	Gordy *et al.* (2017)

36	*Uvulifer* sp.	MK604882	MC	*Tilapia sparrmanii*	Mooi River, South Africa	Hoogendoorn *et al.* (2019)

Supplementary Table S2. Genetic distance of the ITS sequences used in this study. Please refer to Table S1 for the ID of the sequences.

ID	1	2	3	4	5	6	7	8	9	10	11	12	13	14	15	16	17	18	19	20	21	22	23	24	25	26	27	28	29	30	31	32	33	34	35
1		51	51	50	51	50	43	41	131	135	135	137	137	137	137	137	147	147	142	142	142	142	144	142	145	126	123	135	132	132	132	131	131	135	132
2	0.06		0	1	2	14	24	26	133	133	133	139	135	135	135	135	144	144	142	142	142	142	142	140	141	122	119	130	127	127	127	126	126	130	127
3	0.06	0.00		1	2	14	24	26	133	133	133	139	135	135	135	135	144	144	142	142	142	142	142	140	141	122	119	130	127	127	127	126	126	130	127
4	0.05	0.00	0.00		1	13	23	25	132	132	132	138	134	134	134	134	143	143	141	141	141	141	141	139	140	121	118	129	126	126	126	125	125	129	126
5	0.06	0.00	0.00	0.00		14	24	26	133	133	133	139	133	133	133	133	142	142	142	142	142	142	142	140	141	122	119	128	125	125	125	124	124	128	125
6	0.05	0.02	0.02	0.01	0.02		24	25	128	126	126	133	132	132	132	132	141	141	141	141	141	141	142	140	143	122	119	130	127	127	127	126	126	130	127
7	0.05	0.03	0.03	0.02	0.03	0.03		20	127	126	126	131	135	135	135	135	146	146	143	143	143	143	141	139	142	123	120	134	131	131	131	130	130	134	131
8	0.04	0.03	0.03	0.03	0.03	0.03	0.02		121	122	122	126	131	131	131	131	146	146	138	138	138	138	140	140	143	119	118	132	129	129	129	128	128	132	129
9	0.14	0.14	0.14	0.14	0.14	0.14	0.14	0.13		22	22	26	138	138	138	138	153	153	140	140	140	140	149	147	150	131	132	141	140	140	140	141	139	141	140
10	0.15	0.14	0.14	0.14	0.14	0.14	0.14	0.13	0.02		0	26	139	139	139	139	153	153	143	143	143	143	155	153	156	134	135	141	142	142	140	141	139	141	142
11	0.15	0.14	0.14	0.14	0.14	0.14	0.14	0.13	0.02	0.00		26	139	139	139	139	153	153	143	143	143	143	155	153	156	134	135	141	142	142	140	141	139	141	142
12	0.15	0.15	0.15	0.15	0.15	0.14	0.14	0.14	0.03	0.03	0.03		147	147	147	147	160	160	148	148	148	148	161	159	160	134	135	148	145	145	147	148	146	148	145
13	0.15	0.15	0.15	0.14	0.14	0.14	0.15	0.14	0.15	0.15	0.15	0.16		1	1	1	26	26	52	52	52	52	55	55	56	51	55	68	61	61	61	60	62	68	61
14	0.15	0.15	0.15	0.14	0.14	0.14	0.15	0.14	0.15	0.15	0.15	0.16	0.00		0	0	25	25	51	51	51	51	54	54	55	52	56	69	62	62	62	61	63	69	62
15	0.15	0.15	0.15	0.14	0.14	0.14	0.15	0.14	0.15	0.15	0.15	0.16	0.00	0.00		0	25	25	51	51	51	51	54	54	55	52	56	69	62	62	62	61	63	69	62
16	0.15	0.15	0.15	0.14	0.14	0.14	0.15	0.14	0.15	0.15	0.15	0.16	0.00	0.00	0.00		25	25	51	51	51	51	54	54	55	52	56	69	62	62	62	61	63	69	62
17	0.16	0.16	0.16	0.15	0.15	0.15	0.16	0.16	0.17	0.17	0.17	0.17	0.03	0.03	0.03	0.03		0	64	64	64	64	62	60	61	69	69	74	69	69	69	68	70	74	69
18	0.16	0.16	0.16	0.15	0.15	0.15	0.16	0.16	0.17	0.17	0.17	0.17	0.03	0.03	0.03	0.03	0.00		64	64	64	64	62	60	61	69	69	74	69	69	69	68	70	74	69
19	0.15	0.15	0.15	0.15	0.15	0.15	0.15	0.15	0.15	0.15	0.15	0.16	0.06	0.06	0.06	0.06	0.07	0.07		0	0	0	60	59	59	63	64	83	78	78	78	77	77	83	78
20	0.15	0.15	0.15	0.15	0.15	0.15	0.15	0.15	0.15	0.15	0.15	0.16	0.06	0.06	0.06	0.06	0.07	0.07	0.00		0	0	60	59	59	63	64	83	78	78	78	77	77	83	78
21	0.15	0.15	0.15	0.15	0.15	0.15	0.15	0.15	0.15	0.15	0.15	0.16	0.06	0.06	0.06	0.06	0.07	0.07	0.00	0.00		0	60	59	59	63	64	83	78	78	78	77	77	83	78
22	0.15	0.15	0.15	0.15	0.15	0.15	0.15	0.15	0.15	0.15	0.15	0.16	0.06	0.06	0.06	0.06	0.07	0.07	0.00	0.00	0.00		60	59	59	63	64	83	78	78	78	77	77	83	78
23	0.16	0.15	0.15	0.15	0.15	0.15	0.15	0.15	0.16	0.17	0.17	0.17	0.06	0.06	0.06	0.06	0.07	0.07	0.06	0.06	0.06	0.06		4	5	70	70	83	79	79	77	76	76	83	79
24	0.15	0.15	0.15	0.15	0.15	0.15	0.15	0.15	0.16	0.17	0.17	0.17	0.06	0.06	0.06	0.06	0.06	0.06	0.06	0.06	0.06	0.06	0.00		5	70	68	81	77	77	75	74	74	81	77
25	0.16	0.15	0.15	0.15	0.15	0.15	0.15	0.15	0.16	0.17	0.17	0.17	0.06	0.06	0.06	0.06	0.07	0.07	0.06	0.06	0.06	0.06	0.01	0.01		69	67	82	76	76	76	75	75	82	76
26	0.14	0.13	0.13	0.13	0.13	0.13	0.13	0.13	0.14	0.14	0.14	0.14	0.06	0.06	0.06	0.06	0.07	0.07	0.07	0.07	0.07	0.07	0.08	0.08	0.07		10	44	37	37	39	40	40	44	37
27	0.13	0.13	0.13	0.13	0.13	0.13	0.13	0.13	0.14	0.15	0.15	0.15	0.06	0.06	0.06	0.06	0.07	0.07	0.07	0.07	0.07	0.07	0.08	0.07	0.07	0.01		40	35	35	37	38	38	40	35
28	0.15	0.14	0.14	0.14	0.14	0.14	0.14	0.14	0.15	0.15	0.15	0.16	0.07	0.07	0.07	0.07	0.08	0.08	0.09	0.09	0.09	0.09	0.09	0.09	0.09	0.05	0.04		17	18	15	16	16	0	17
29	0.14	0.14	0.14	0.14	0.13	0.14	0.14	0.14	0.15	0.15	0.15	0.16	0.07	0.07	0.07	0.07	0.07	0.07	0.08	0.08	0.08	0.08	0.09	0.08	0.08	0.04	0.04	0.02		3	6	7	7	17	0
30	0.14	0.14	0.14	0.14	0.13	0.14	0.14	0.14	0.15	0.15	0.15	0.16	0.07	0.07	0.07	0.07	0.07	0.07	0.08	0.08	0.08	0.08	0.09	0.08	0.08	0.04	0.04	0.02	0.00		7	8	8	18	3
31	0.14	0.14	0.14	0.14	0.13	0.14	0.14	0.14	0.15	0.15	0.15	0.16	0.07	0.07	0.07	0.07	0.07	0.07	0.08	0.08	0.08	0.08	0.08	0.08	0.08	0.04	0.04	0.02	0.01	0.01		1	3	15	6

32	0.14	0.14	0.14	0.13	0.13	0.14	0.14	0.14	0.15	0.15	0.15	0.16	0.06	0.07	0.07	0.07	0.07	0.07	0.08	0.08	0.08	0.08	0.08	0.08	0.08	0.04	0.04	0.02	0.01	0.01	0.00		4	16	7
33	0.14	0.14	0.14	0.13	0.13	0.14	0.14	0.14	0.15	0.15	0.15	0.16	0.07	0.07	0.07	0.07	0.08	0.08	0.08	0.08	0.08	0.08	0.08	0.08	0.08	0.04	0.04	0.02	0.01	0.01	0.00	0.00		16	7
34	0.15	0.14	0.14	0.14	0.14	0.14	0.14	0.14	0.15	0.15	0.15	0.16	0.07	0.07	0.07	0.07	0.08	0.08	0.09	0.09	0.09	0.09	0.09	0.09	0.09	0.05	0.04	0.00	0.02	0.02	0.02	0.02	0.02		17
35	0.14	0.14	0.14	0.14	0.13	0.14	0.14	0.14	0.15	0.15	0.15	0.16	0.07	0.07	0.07	0.07	0.07	0.07	0.08	0.08	0.08	0.08	0.09	0.08	0.08	0.04	0.04	0.02	0.00	0.00	0.01	0.01	0.01	0.02	
